# Pilot Randomised Evaluation of Singing in Dementia (PRESIDE): protocol for a two-arm, parallel-group randomised controlled feasibility study with waiting-list control

**DOI:** 10.1186/s40814-020-00759-y

**Published:** 2021-01-07

**Authors:** Becky Dowson, Justine Schneider, Boliang Guo, Philip M. Bath, Orii McDermott, Lee J. Haywood, Martin Orrell

**Affiliations:** 1 Institute of Mental Health, University of Nottingham, Jubilee Campus, Triumph Road, Nottingham, NG7 2TU UK; 2 Stroke Trials Unit, Division of Clinical Neuroscience, University of Nottingham, Clinical Sciences Building, Nottingham, PB NG5 1 UK

**Keywords:** Dementia, Carers, Community, Singing, Music, Mental health, Social isolation, Well-being, Psychosocial interventions, Feasibility study

## Abstract

**Background:**

As the number of people living with dementia grows, so does the need to provide them with adequate psychosocial support. Many people with dementia live at home with family carers, who also require social and emotional support to cope with their role. Community group singing has received attention for its potential to support people with dementia and their carers. It is postulated that singing can improve cognitive function, strengthen the bonds between care partners and help to establish social support networks. However, there is a lack of rigorous evidence of singing’s benefits for this population. This study aims to test the feasibility of a randomised controlled trial of community singing in dementia, to pave the way for a larger, conclusive study.

**Methods:**

The PRESIDE study is designed as a two-arm, parallel-group randomised trial with a waiting list control. Dyads consisting of a person with dementia (*n* = 80) and their carer (*n* = 80) will be recruited. Each dyad will be randomised either to attend 10 weeks of community group singing sessions straight away or to wait for 3 months before attending the sessions. The singing sessions will be led by experienced professional musicians and will last about 90 min, including time for socialising. The primary outcome of this study is the attainment of feasibility criteria around recruitment, retention and the acceptability of the waiting list control. Secondary outcomes include the quality of life, mood, cognition, and musical engagement of the person with dementia, and quality of life, mood, and experiences/challenges of the carer. These data will be collected during home visits at baseline, and 3 and 6 months post-baseline.

**Discussion:**

Despite growing public interest in the positive effects of singing, and encouraging findings from qualitative and non-randomised quantitative studies, there is a lack of rigorous evidence. This is the first randomised controlled trial of community group singing for people with dementia in Europe, to our knowledge. If the results favour a full trial, conclusively demonstrating the effectiveness of group singing could positively affect the opportunities available to community-dwelling people with dementia and their carers.

**Trial registration:**

Unique identification number in ISRCTN registry: ISRCTN10201482. Date registered: 12 May 2020

## Introduction

### Background and rationale

As our population ages, the number of people living with dementia is set to increase, with an estimated 2 million people predicted to have the condition in the UK by 2050. Although dementia is progressive, people may live for many years after diagnosis. Residential care is an option for those with higher needs, but the majority (61%) of people with dementia live in the community, many supported by family carers [1]. People with dementia are at greater risk of loneliness and mental health problems than the general population [2, 3]. Although many family carers find their role rewarding, caring responsibilities can incur a significant psychological and physical strain. Unless they are well resourced and supported, carers may find themselves at heightened risk of depression and anxiety, and consequently with poor quality of life [4]. If caregivers experience unalleviated and overwhelming challenges in their role, this may result in earlier admission to residential care for the person with dementia, leading to a lower quality of life for them and increased pressure on social care services [5].

Consequently, there is a need for psychosocial interventions, which support people with dementia and their carers to live well with the condition for as long as possible. One such intervention is cognitive stimulation therapy (CST), which can improve cognition and quality of life for people with dementia and is recommended by the National Institute for Health and Care Excellence (NICE) for all people with mild or moderate dementia [6, 7]. Community group singing is growing in popularity as an activity to support the mental well-being of people with dementia and their carers. There is evidence from neuropsychology that singing provides cognitive stimulation through multiple effects on the brain, but that response to singing is mediated by clinical and demographic factors [8]. A randomised trial compared the effects of singing, listening to music and usual care for person with dementia/carer dyads, and found that both singing and listening to music improved mood, orientation and remote episodic memory, whilst singing also improved carer well-being [9]. Qualitative evidence indicates that singing for people with dementia has a positive effect on the relationship between couples when one partner has a diagnosis of dementia [10] whilst a study of group singing in the general population has indicated that it may accelerate social bonding [11]. Participants at singing groups for those with dementia have also reported that they find the support network provided by meeting other people in a similar situation very helpful [12]. Meanwhile, there is evidence from a randomised controlled trial that group singing is a cost-effective way of improving quality of life and reducing anxiety and depression for people over the age of 60 [13].

Despite the growing interest in group singing for people with dementia, there is a lack of conclusive evidence about its effects. To date, no published large-scale, randomised study has evaluated group singing for people with dementia and their carers in the community. A study of this kind has recently been completed in Australia (Australian and New Zealand Clinical Trials Registry (ANZCTR) ID: ACTRN12617001513303) and the results are pending publication (this study has singing groups facilitated by music therapists whilst the facilitators in the present study are community musicians, so this will make an interesting point for comparison). Evidence from smaller studies is variable; several studies found no change on standardised measures of quality of life, mood, or neuropsychiatric symptoms of dementia, but reported favourable qualitative findings [14–16]. In a small pre-post study of group singing which showed improvements in quality of life, self-esteem and social support for people with dementia and their carers, the authors call for an evidence base to demonstrate the psychological benefits and potential for improvement in cognitive function which they believe group singing affords [17]. Therefore, there is an identified need for rigorous RCT evidence to isolate the effects of group singing for people with dementia and their carers. Such evidence could inform health and social care policy, help to secure additional funding for group singing and potentially reduce the pressure on health and social care services by improving support for people living with dementia in the community and their carers.

### Study objectives

The primary objective of the PRESIDE study is to test the protocol for a two-arm, parallel-group superiority randomised trial of community singing in dementia with a waiting-list control design. The relevant indicators are recruitment, retention, and the acceptability of the trial designs to the participants, including the waiting-list control. The rationale for the waiting list design is that a control group, which only receives care as usual, would be likely to experience resentful demoralisation with attendant drop-out. If the results of this feasibility study are favourable, a full-scale trial would aim to recruit 360 dyads, sufficient to identify a moderate effect size on quality of life (0.3) [13, 18].

The secondary objective of the study is to explore the impact of group singing on people with dementia and their carers, using quantitative and qualitative methods. It is anticipated that this data will indicate suitable outcome measures for the full trial, and improve our understanding of the mechanisms of action at play in group singing for people with dementia.

## Methods

### Study design

This feasibility study is designed as a two-arm, parallel-group randomised controlled trial of community singing for people with dementia and their carers, with a waiting-list control group. The study will aim to recruit 80 dyads each consisting of a person with dementia and their carer. About half of the sample, selected at random, will be invited to attend a singing group, whilst the other half will be asked to wait for 3 months before being invited to attend a group. Outcome data will be collected at baseline (time 0), 3 months after baseline (time 1), and 6 months after baseline (time 2).

### Setting

The singing groups created for the study will take place in community settings in Nottinghamshire, UK, and data collection will take place during visits to participants at home. The venues selected will be chosen for their suitability for holding singing group sessions; this means well-lit, attractive spaces with good acoustics that are large enough to comfortably accommodate up to 50 people. Further considerations include the provision of accessible facilities and a kitchen for refreshments, adequate car parking and a reliable and frequent public transport service nearby. We will focus on two geographical areas and will aim to recruit half the intended target of 80 dyads in each of these areas. Recruitment will be optimised by selecting venues in geographical areas that are believed to have the highest levels of need in terms of the provision of recreational singing for people with dementia.

### Inclusion and exclusion criteria

To be included in the study, participants with dementia must

- Be aged 18 or over, and diagnosed with dementia, which is at a mild or moderate level
- Have a carer who spends at least 2 h per week with them
- Be able to give informed consent at the start of the study
- Be able to speak and understand English^1^
- Be willing in principle to join a singing group and attend weekly

Participants with dementia will be excluded if

- They lack capacity to give informed consent
- They are already attending a singing group or have done so in the past 6 weeks (other than religious services)
- They have a significant hearing impairment
- They are already participating in any other interventional study
- They have a history of severe mental illness or drug/alcohol addiction

Carers can be included if they are

- Able to speak and understand English
- Willing in principle to attend the group weekly
- Able to give informed consent

Relationships between people with dementia and their carers may be that of spouses/partners, parent/child or other family members, or friends. In certain cases, professional caregivers may be part of the study if no family member or friend is available to accompany a person with dementia to the sessions and if the same professional caregiver is available each week. The nature of the relationship between the dyads does not matter, but it is important that the same carer attends the sessions throughout the study.

### Intervention

The intervention in this study is attendance at weekly singing group sessions for 10 weeks. Each session will include approximately 60 min of warm-up activities and singing, with about 30 min for refreshments and socialising before or after the session. The sessions will be run jointly by two professional community musicians who have extensive experience of running similar singing groups together. The format of the sessions includes a full physical and vocal warm-up, followed by singing familiar songs accompanied by guitar. Themed songbooks will be provided for participants; each week a different songbook will be selected by the facilitators, so each session will have a different theme. Themes might include songs about the weather, from a particular era, or relating to the current season (e.g. Christmas, summer). Singing group participants will be encouraged to choose their favourite songs from the books for the whole group to sing. The facilitators’ approach will emphasise that the aim of the group is to sing for fun and enjoyment, and that the participants’ musical skills or singing voices are not being assessed or judged.

The content of each session will be fully documented, and three out of every ten sessions will be video recorded in order to check that the intervention is delivered consistently. Attendance at singing sessions will be monitored and recorded. Participants will be free to miss individual sessions or withdraw from the singing group entirely as they wish, and they do not need to give a reason.

Since each singing group can accommodate up to 20 dyads, we propose to run four groups in total in two different geographical areas. The first two groups will be set up for those allocated to the intervention group; after the 3 month waiting period, we will start another two groups for those in the waiting-list control group. There is provision for those in the intervention group to continue to attend the singing groups after the initial 10-week period, if they wish to. This is for ethical reasons and to facilitate embedding the groups into the local community, as discussed later in the “Ethics and dissemination” section. Continued attendance by participants in the intervention group will not compromise the primary objectives of recruitment, retention and acceptability, and its effects on the 6-month data collection are considered an acceptable trade-off.

### Control condition

Participants who are allocated to the waiting-list control group will receive care as usual during the 3 months that they will be required to wait before beginning to attend a singing group. They will be requested to abstain from attending other organised group singing activities during this time, with the exception of religious services.

### Endpoints

Since PRESIDE is a feasibility study, the endpoint is the attainment of the feasibility criteria at 6 months. The proposed criteria for proceeding to a full trial are 70% recruitment; 70% retention of participants over 3 months; and acceptability of the control condition demonstrated by 70% of the waiting-list participants starting singing groups after being on the waiting list. The acceptability of the control condition is considered to be the most important of these three criteria since this ensures the heterogeneity of the sample. The other feasibility criteria are considered negotiable.

An additional endpoint for PRESIDE is the measurement of quality of life, activities of daily living, mood, musical engagement and cognition of people with dementia, and the measurement of quality of life, mood, caring relationship and experience of caring for the carers of the people with dementia. Each dyad’s use of health social care services and resources will also be documented. Additionally, qualitative data will be collected using interviews and participant observation, in order to better understand the experiences of dyads who attend the singing groups.

### Participant timeline in study

**Fig. 1:**
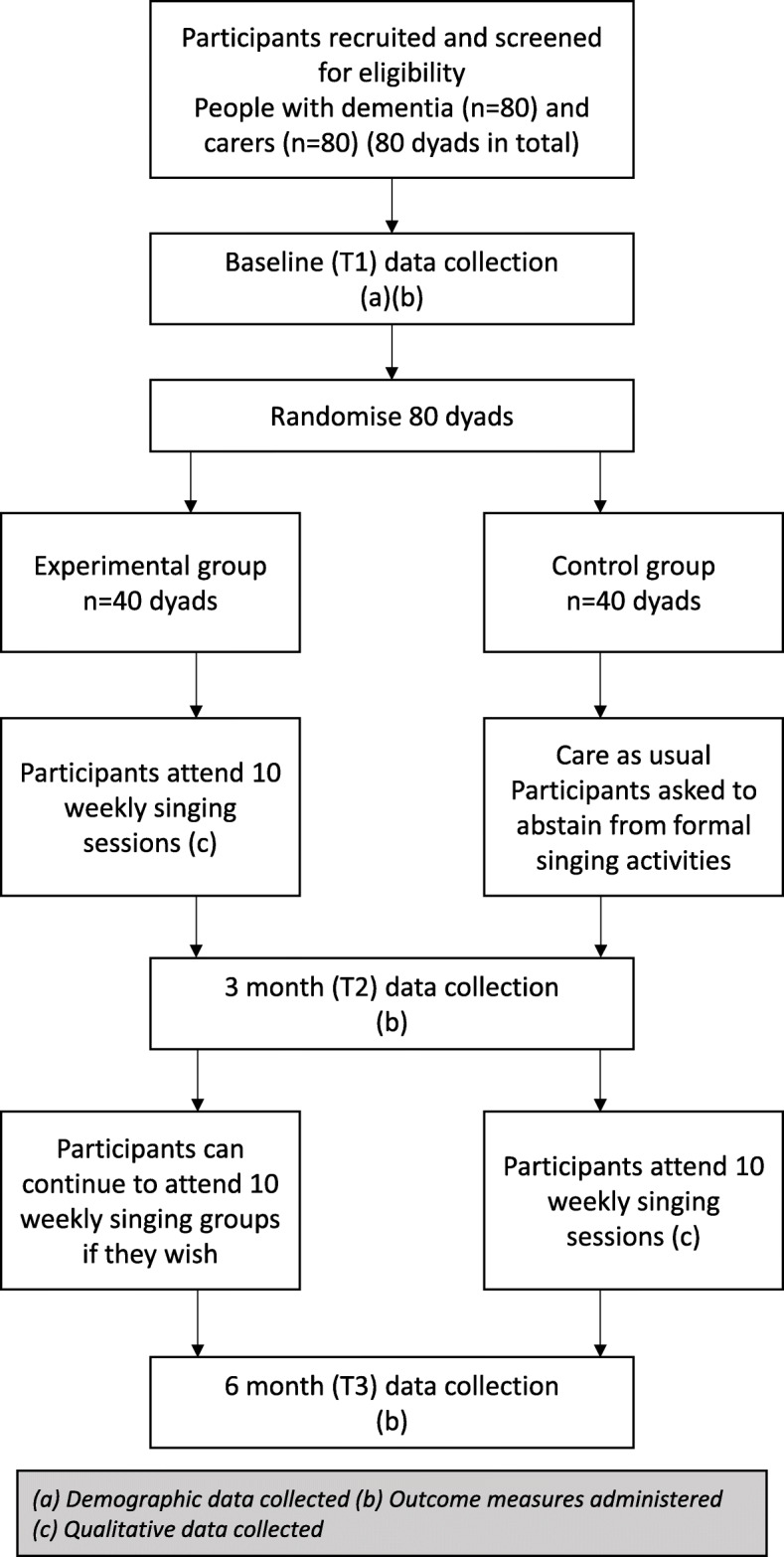
Participant timeline in study

**Fig. 2:**
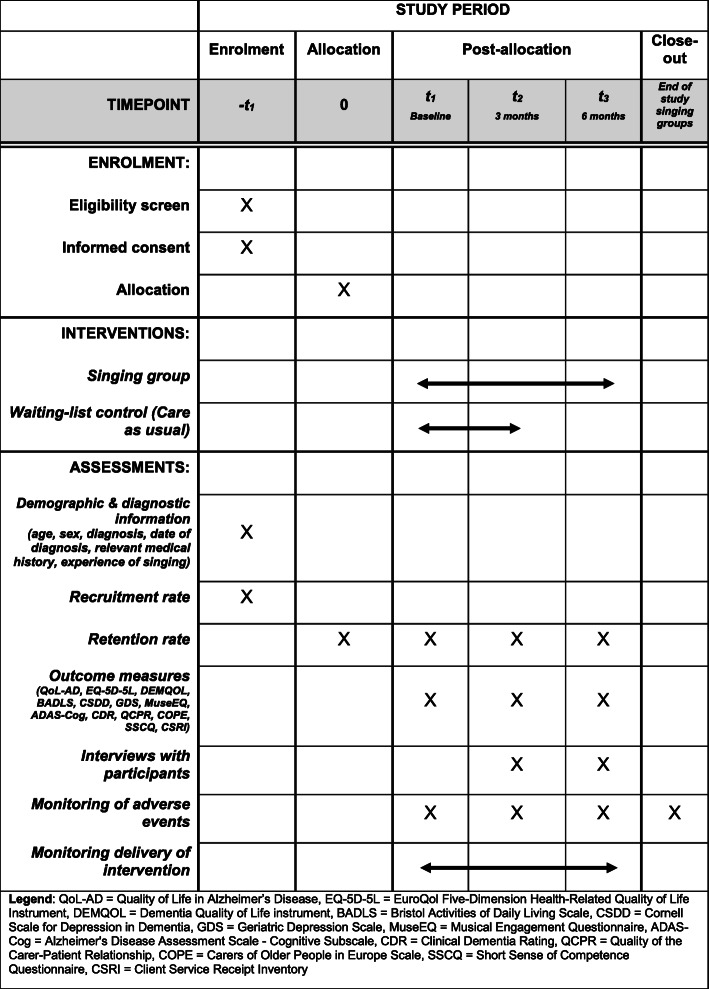
Schedule of enrolment, intervention and assessment for PRESIDE

Figure 1 shows the timeline of participants in the study, whilst Fig. 2 gives the schedule of assessment and enrolment for the study. Since we need to recruit a sufficient number of participating dyads before the singing groups can begin, there will be a delay between recruitment and beginning the singing group or waiting list control for participants who are recruited early. We will not collect baseline data or randomise participants until the singing groups are just about to start.

### Sample size

As PRESIDE is a feasibility study with two arms, it is suggested 40 dyads per arm will provide enough information to inform future, definitive RCT design and implementation [19]. Therefore the recruitment target is 80 patient-carer dyads in total in the current study.

### Allocation and blinding

Participants will be recruited in pairs consisting of a person with dementia and a carer. They will be randomised in pairs to either the intervention group or the waiting-list control group. Randomisation will be stratified by sex and Clinical Dementia Rating (CDR). Randomisation will be carried out using a web application created by the trial programmer at the University of Nottingham Stroke Trials Unit, using web and database servers provided by University of Nottingham Information Services (IS). The researcher (BD) will use this web application to randomise each participant. The system will hide information relating to the intervention groups from other users until explicitly granted access by the trial programmer, for example, when a statistician requires unblinded data.

Dyads will be randomly allocated to intervention or waiting-list control groups by the web-based system. BD will collect baseline data prior to randomisation, and so will be unaware of allocation until the initial data collection is complete. Thereafter, both the researcher (BD) and the participants will be aware of the group allocation, along with those delivering the sessions. Due to the nature of the intervention and control groups, it is not feasible to blind participants to group allocation; they will know whether they are attending singing group sessions. The research team will be split into two groups, which will define their access to data and to group allocation. The data management group will be responsible for the data collection and running of the study, and will not be blinded to group allocation once baseline data collection has taken place. The data analysis group will be responsible for conducting the analysis, and will remain blinded to group allocation for the duration of the study. The data analysis group will not have any contact with study participants.

Follow-up data collection will be undertaken by researchers who are blinded to group allocation. However, since the participants will know their own group allocation, there is a possibility that they will reveal this during the follow-up data collection. Unblinding of outcome assessors which occurs in this way will be recorded. All researchers undertaking data collection will have been fully trained in the administration of these instruments.

### Recruitment

We will recruit for the study through various channels in order to maximise the chances of reaching as many people with dementia as possible. We will recruit through NHS Memory Assessment Services by publicising the study to people who have recently been diagnosed with dementia. We also have agreement from the Alzheimer’s Society to advertise the study to people who are currently on the waiting list in the area for Singing for the Brain sessions. We will harness the support of local community organisations, charities and the local press, and will also use targeted social media advertising. Recruitment will also be undertaken using the online platform “Join Dementia Research”.

### Data collection

#### Quantitative data

Outcome measures will be administered to participants during home visits arranged at times convenient to them. Each visit is expected to take up to 2 h. Baseline data collection will be conducted by BD, and follow-up data collection by blinded researchers. All researchers undertaking data collection will have been fully trained in the administration of these instruments.

The following outcome measures have been selected for use in the study. These are summarised in Table 1, which also shows whether each measure corresponds to the person with dementia, the carer, or both, as well as who completes the measure (the person with dementia, or the carer, either self- or proxy-rating). All outcome measures will be administered at each of the three-time points. Additionally, demographic data will be collected from people with dementia (age, sex, diagnosis, date of diagnosis, ethnicity, education, musical/singing experience) and from carers (age, sex, ethnicity, education, musical/singing experience) at baseline. Measures will be completed by the person with dementia themselves and/or via a proxy rating by the carer. The carer will complete outcome measures about their own mood, quality of life and experience of caring. Some of these measures will be administered by interview and some will be self-completed by the carer.

**Table 1:** Summary of outcome measures

Instrument name	Domain measured	Participant		Respondent		
		Person with dementia	Carer	Person with dementia	Carer (proxy)	Carer (self)
QoL-AD	Quality of life	x		x	x	
EQ-5D-5L	Health-related quality of life	x	x	x		x
DEMQOL	Quality of life	x		x		
BADLS	Activities of daily living	x			x	
CSDD	Depression	x		x	x	
GDS	Depression	x	x	x		x
MuseEQ	Musical engagement	x		x		
ADAS-Cog	Cognition	x		x		
CDR	Dementia severity	x		x	x	
QCPR	Caring relationship		x			x
COPE	Experiences/consequences of caring		x			x
SSCQ	Experiences of caring		x			x
CSRI	Use of health/social care	x	x		x	x

A large number of outcome measures are included in this study, in order to assess which of them might be most suitable for future use in a full trial. We recognise that data collection may be time-consuming and tiring for participants, and consequently missing data and incomplete measures are expected in some cases. The order and format of the administration of outcome measures is given in Table 2. This order has been chosen so that instruments measuring similar domains are separated, to maximise the range of information included in incomplete data sets. The interview with the carer will take place first, and subsequently the person with dementia will be interviewed whilst the carer does the self-completed outcome measures.

**Table 2:** Order and format of outcome measure administration

Person with dementia (by interview)	Carer	
	By interview	Self-complete
1. QoL-AD 2. GDS 3. CDR 4. MuseEQ 5. ADAS-Cog 6. EQ-5D-5L 7. CSDD 8. DEMQOL	1. QoL-AD proxy 2. GDS 3. CDR 4. QCPR 5. CSRI	1. COPE 2. SSQC 3. EQ-5D-5L 4. BADLS

##### Quality of life—Alzheimer’s disease scale

This 13-item measure asks respondents to rate various aspects of the person with dementia’s life as poor, fair, good or excellent [20]. It will be completed by participants with dementia, and a proxy rating will also be provided by the carer. Each item is scored on a scale from 1 to 4, so the measure yields a total score out of 52, where a higher score indicates a higher quality of life.

##### EQ-5D-5L

This instrument comprises five questions about health-related quality of life, and a visual analogue scale [21–23]. It will be completed by participants with dementia and carers. The results will be used to calculate Quality-Adjusted Life Years (QALYs) for all participants. The first five questions are scored on a five-point scale and generate a five-digit health state for each participant (e.g. a score of 12345 would indicate no problems with mobility, slight problems with self-care, moderate problems doing usual activities, severe pain or discomfort, and extreme anxiety or depression). The visual analogue scale is the final item of the EQ-5D-5L and asks respondents to rate their current health as a point on a line. This gives a score out of 100, where a higher score indicates a higher perceived level of overall health.

##### Dementia quality of life

This scale asks the person with dementia to rate their quality of life by responding to 29 questions about their feelings, memory and everyday life [24]. The respondent selects ‘a lot’, ‘quite a bit’, ‘a little’ or ‘not at alL’ in response to each question. Each question is scored on a scale from 1 to 4, giving a total score out of 116 where a higher score indicates a higher quality of life.

##### Bristol activities of daily living scale

The 20-item scale presents the respondent with various descriptions of performance at activities such as cooking, hobbies and personal care, and asks them to rate the person with dementia’s current level of performance [25]. It will be completed as a proxy rating by the carer. Each items is scored on a scale of 0 to 3, and gives a total score out of 60, where a higher score means a higher level of impairment.

##### Cornell scale for depression in dementia

The 19-item scale assesses symptoms of depression in people with dementia and their severity [26]. It is completed through interviews conducted with the person with dementia and the carer separately, with the interviewer assigning a score based on the severity of symptoms within the past week. For each indicator of depression, absent symptoms score 0, mild or intermittent symptoms score 1, and severe symptoms score 2. The total score is out of 38, where scores above 10 indicate probable major depression, and scores above 18 indicate definite major depression.

##### Geriatric depression scale

The 15-item version of this scale presents the respondent with ‘yes/no’ questions about how they have been feeling over the past two weeks [27]. It will be completed by people with dementia and carers. Each answer indicative of depression scores one point. A score of 5 or higher is suggestive of a depressive disorder, whilst a score of 10 or higher is indicative of a probable depressive disorder.

##### Musical engagement questionnaire

This scale has 35 items that ask the participant how they have used and engaged with music over the past month [28]. It will be completed by people with dementia. The scale consists of statements such as ‘I play a song over and over if I like it’ and the respondent indicates the extent to which they agree with the statement on a five-point scale, scored from 1 to 5. The total score is out of 175, and a higher score indicates a higher level of musical engagement.

##### ADAS-Cog

The measure assesses a number of facets of cognitive performance including memory, verbal skills, motor skills, and orientation [29, 30]. It will be completed by the person with dementia. The tests include memorising a list of words, naming familiar objects as they are presented, copying drawn shapes and following verbal instructions. The interviewer also rates the participant on their verbal fluency, comprehension and ability to concentrate. The total possible score is 70, with higher scores indicating higher levels of cognitive impairment.

##### Clinical dementia rating

This instrument gathers data through semi-structured interviews conducted with the carer and the person with dementia [31]. The interviewer uses this data to rate the severity and stage of dementia across six different domains: memory, orientation, judgement/problem solving, community affairs and home/hobbies. The rating of each of these domains is used to generate an overall CDR score which indicates the level of impairment present: 0 (none), 0.5 (questionable), 1 (mild), 2 (moderate) or 3 (severe).

##### Quality of the carer-patient relationship scale

The 14-item measure invites the respondent to state their level of agreement with questions about their relationship with the person for whom they care [32]. It will be completed by carers. The responses are scored from 1 to 5, and the total score is out of 70, with a higher score indicating higher perceived relationship quality.

##### Carers of older people in Europe scale

The 15-item scale asks the carer about their experiences of caregiving and its consequences for their physical and mental health [33]. The responses are scored from 1 (never) to 4 (always). The 15 questions are presented together, but when total scores are calculated they are divided into three subscales which are treated separately in analysis: negative impact of caregiving, positive value of caregiving and quality of support. The precedent for the use of these subscales in analysis is set in one of the paper describing the original development of COPE [34].

##### Short sense of competence questionnaire

The SSCQ is derived from the Zarit Burden Inventory and evaluates the carer’s experience of caring across seven items [35]. The carer indicates how strongly they agree with the statements in the scale and each item is scored from 1 to 5. A higher score indicates a higher perceived sense of competence.

##### Client service receipt inventory

The CSRI is a tool to measure consumption of services with a view to estimating their costs for economic analysis [36]. It will be administered in a form adapted to the typical activities of people with dementia and their carers, in order to measure the use of health and social carer resources by each participant. This instrument will be completed by the carer.

#### Qualitative data

Previous research into group singing for people with dementia has shown that quantitative measures may not capture the benefits perceived by participants [14, 37]. Qualitative data concerning participants’ experience of the singing groups will be collected during the study using participant observation. BD will conduct structured in-person observations of 3-4 opportunistically selected dyads in each singing group, including both intervention and control group participants. Each dyad will be the focus of observations three times: in one early session, in one session towards the middle of the group’s course, and in one later session. Detailed notes on the observations will be made after the sessions.

We will also collect qualitative data from a sub-sample of participants through semi-structured interviews. We will aim to interview 10 dyads in total, 12.5% of the 80-dyad intended sample. Dyads will be selected for interview opportunistically and those who do not wish to be interviewed will be free to decline. We will interview at least one dyad from each singing group and will aim to spread the sample evenly between the groups. Interviews will last 30-45 min, and each dyad will be interviewed together where possible. The interview guide will focus on understanding participants’ experience of taking part in the group. Interviews will be audio recorded if possible; if participants decline to be recorded, we will seek their permission to make written notes during the interviews.

### Data management

Each participant will be assigned a trial identity code number, for use on case report forms (CRFs) and in the database. The trial documents and database will also use participants’ initials, sex and date of birth. Data will be recorded for each participant using paper CRFs. The data from these forms will be entered into the trial database and stored electronically. Access to the database will be password protected and restricted to trial personnel, who will each have a unique login code and PIN. The paper CRFs will be stored in a locked cupboard on the University of Nottingham premises. Consent forms (which contain participants’ real names) will be stored separately in a different locked cupboard. Participant’s names and contact information will be securely stored on a separate part of the database system, not associated with their trial data.

Any video or audio recordings taken during data collection will be transferred daily from the recording device to password-protected encrypted drives on University of Nottingham computers. They will then be deleted from the recording device.

Data will be stored securely for 7 years after the last person has been recruited. All data will be analysed on University of Nottingham computers and backed up to the University of Nottingham servers. Only members of the research team authorised by the data custodian will have access to the research data.

### Data coding and analysis

#### Statistical analysis of quantitative data

Quantitative data analysis will be conducted by the study statistician (BG), using the software package Stata 16. The data analysis will be mainly descriptive [38]. All measures will be summarised with mean and standard deviation (S.D.) for normally distributed variables, median and inter-quartile range (IQR) for skewed variables, and frequency (%) for response of categorical variables, by intervention arm across follow-up time. Analysis of Co-variance (ANCOVA) will be performed to quantify treatment effect size and its precision by means of regression modelling with baseline measures as covariate, and to derive the change from baseline score for each arm. Due to the non-independence of patient-carer dyad measures and with data being measured repeatedly, multivariate, multilevel modelling will be performed to account for the association between the same kind of outcome measure of patient-carer data and repeated data. We will calculate recruitment rate, retention rate and attrition rate, exploring patterns of missing data and drop out from the intervention group and the waiting list control group. All results will be used to inform future definitive trial design.

#### Analysis of qualitative data

Recordings/notes from interviews and observational notes will be transcribed as necessary and analysed using NVivo 12. The technique for analysis will be the ‘general inductive approach’ in which the coding is largely guided by evaluation objectives [39]. The evaluation objectives in this case will be the feasibility of the study design and the experiences of all participants who attend a singing group.

BD will undertake the first stage of the analysis and will assign first-order codes to the data. Through discussion with the rest of the research team, second-order codes will then be developed. In order to enhance the validity of the coding process, a sample of transcripts and notes will be coded by another member of the research team, and any disagreements between coders will be discussed.

### Data monitoring

A trial Data Management and Ethics Committee (DMEC) will be formed, consisting of members who are not part of the University of Nottingham. All the members of the DMEC will have experience of running clinical trials, and the group will include a statistician, an academic and a clinician. The Chief Investigators (JS & MO) will be the data custodians.

### Adverse events

The occurrence of an adverse event as a result of participation in this study is not expected due to the nature of the intervention, which is a non-clinical intervention based on social music-making and has no known side effects. However, in order to provide further assurance of the safety of the intervention, data on Severe Adverse Events (SAEs) will be collected for people with dementia and their carers during follow-up data collection at 3 and 6 months. Additionally, attendance at the group will be monitored by the singing group facilitators and the research associate, so any SAE which occurs amongst the group members will be recorded. These include hospitalisation, extension of hospitalisation, other serious medical events and death. Such SAEs might conceivably be related to participation in the intervention if they were associated with falls, emotional arousal or depression.

## Ethics and dissemination

### Ethical issues and approval

The application for ethical approval for PRESIDE was submitted to the Health Research Authority’s Social Care Research Ethics Committee (SCREC) and received a favourable opinion after some minor revisions. Ethical approval for the study was granted in January 2020 and the study entered the recruitment phase.

Due to lower than anticipated recruitment figures and advice from the PPI group, a substantial amendment to the ethical application was submitted to the SCREC in March 2020. This amendment removed a previous inclusion criterion that stipulated that people with dementia should have received their diagnosis within the past 12 months, thus opening the study to anyone with a diagnosis of dementia regardless of when it was made. This amendment was approved by the SCREC in April 2020.

A major ethical issue for this trial is that it provides the participants with the chance to attend a community singing group which they may find supportive, helpful or valuable. Therefore, it is important to consider what will happen at the end of the trial, as the sudden cessation of the singing groups could be harmful or upsetting to participants. Although PRESIDE does not have the resources to continue to run these groups indefinitely, we aim to work with local organisations and charities to embed the singing groups into the local community and to make them sustainable in the longer term. This process will be guided by the opinions and wishes of the singing group participants.

### Consent

All participants will provide written informed consent before they participate in any study activities. Written information sheets will be provided for people with dementia and their carers. An easy-read information booklet will also be provided to facilitate the understanding of people with dementia. Potential participants who contact the study team will be provided with a copy of the participant information sheets. The researcher will visit the potential study participants and will go through the information sheets with them, answering any questions that they have. If they agree to participate in the study, and the researcher is satisfied that they have capacity to provide informed consent to do so, they will be asked to sign a consent form.

A person with dementia who does not have capacity to consent to participate at the point of enrolment will be excluded from the study. However, due to the progressive nature of dementia, participants with dementia may lose capacity over the course of the study. Capacity will be reassessed periodically throughout the course of the study. If a person with dementia is judged to have lost capacity in this regard, the carer who is taking part in the study with them will be asked to act as consultee, and to make a judgement about whether the person they care for would have wanted to continue to take part. The consultee will be provided with an information sheet advising them about the study and the role of consultee. If they judge that the person with dementia would have wanted to continue to take part, the consultee will sign a declaration form to indicate that this is their opinion. The consent of carers will be treated in the same way; if a carer loses capacity to consent during the course of the study, a consultee will be sought for them.

Any participants who decide not to take part or who are excluded because they lack capacity will be signposted as far as possible to other community-based musical or creative groups that they may be able to access.

### Confidentiality

Individual participant medical information obtained during this study is considered confidential and will not be disclosed to third parties except for reasons of data monitoring and auditing, or where such disclosure is considered necessary for the health or wellbeing of the participant. Confidentiality will be ensured by using participant identification codes to refer to stored data rather than real names. When findings from the study are disseminated or published, individual participants will not be identifiable from the reports.

### Dissemination

A journal paper describing the findings of the feasibility study will be submitted for publication. The results of the study will also be reported to at least one major dementia conference. Participants in the study will be sent a summary of results in the post if they wish. If the results of the study suggest that a full trial is feasible, a protocol will be developed based on the results and submitted to a suitable research funding body.

### Public involvement

A Public Participation and Involvement (PPI) group has been set up for the study, and a convenor for the group appointed. The group comprises individuals who either have lived personal experience of dementia or experience of caring for someone with dementia. The scope of the PPI group’s role is as follows:

- Advising on issues around the acceptability of the research process (e.g. recruitment, data collection)
- Refinement of research materials (e.g. participant information sheets)
- Scrutiny of public-facing materials
- Advocacy for the research
- Advising about recruitment methods
- Helping with dissemination

Any suggestions, advice and feedback from the PPI group will be implemented in this study where possible or will be used to inform the design of future research.

PPI meetings take place every 3 months, and delegates from the PPI group attend Project Management Group meetings. The PPI group members are recompensed for their time and travel expenses. During the COVID-19 disruption, the PPI group continues to meet and to work with the research team on related projects.

## Discussion

Following ethical approval in January 2020, the study entered recruitment, aiming to start the first singing group in late April. This would require approximately 40 dyads to have been recruited, in order to randomise about half to the singing group and half to the waiting-list control. There was a reasonably high level of interest in the study but recruitment was slower than had been hoped for. It was felt that the requirement for participants to have been diagnosed within the past twelve months was hindering recruitment, since quite a lot of interest had been expressed by people with dementia who had been diagnosed for longer and therefore were not eligible. Consequently, a major amendment was submitted to the SCREC in order to change the inclusion criteria. It is hoped that this change will increase the rate of recruitment to the study.

In March 2020, recruitment for PRESIDE was paused due to the COVID-19 pandemic. Although the immediate future is unclear, we hope that the initial groundwork which we have done will help us to recruit more rapidly for the study once it is deemed safe to restart. In the meantime, our priority is to maintain an online presence and publicise the study in a way which will support recruitment in the future.

## Data Availability

Not applicable

## Abbreviations

ANCOVA: Analysis of co-variance
BADLS: Bristol activities of daily living scale
CDR: Clinical dementia rating
COPE: Carers of older people in Europe
CSDD: Cornell Scale for depression in dementia
CSRI: Client service receipt inventory
DEMQOL: Dementia quality of life instrument
DMEC: Data monitoring and ethics committee
GDS: Geriatric Depression Scale
IQR: Inter-quartile range
MuseEQ: Musical engagement questionnaire
NICE: National institute for health and care excellence
PPI: Public participation and involvement
QoL-AD: Quality of life in Alzheimer’s disease
QCPR: Quality of the carer-patient relationship
SCREC: Social care research ethics committee
SSCQ: Short sense of competence questionnaire
SAE: Severe adverse event
SD: Standard deviation

## Footnotes

1 Participants with aphasia or other speech problems will be included unless these were so severe that the participant could not answer the questionnaires, or communicate their answers in an alternative way.
